# Phenol-Grafted Alginate Sulfate Hydrogel as an Injectable FGF-2 Carrier

**DOI:** 10.3390/gels8120818

**Published:** 2022-12-12

**Authors:** Ryota Goto, Masaki Nakahata, Shinji Sakai

**Affiliations:** 1 Department of Materials Engineering Science, Graduate School of Engineering Science, Osaka University, Toyonaka 560-8531, Japan; 2 Department of Macromolecular Science, Graduate School of Science, Osaka University, Toyonaka 560-0043, Japan

**Keywords:** FGF-2, hydrogel, tissue engineering, injectable, drug delivery system, alginate, HRP

## Abstract

In the field of tissue engineering, fibroblast growth factor-2 (FGF-2) effectively regenerates damaged tissue and restores its biological function. However, FGF-2 readily diffuses and degrades under physiological conditions. Therefore, methods for the sustained and localized delivery of FGF-2 are needed. Drug delivery systems using hydrogels as carriers have attracted significant interest. Injectable hydrogels with an affinity for FGF-2 are candidates for FGF-2 delivery systems. In this study, we fabricated a hydrogel from phenol-grafted alginate sulfate (AlgS-Ph) and investigated its application to the delivery of FGF-2. The hydrogel was prepared under mild conditions via horseradish peroxidase (HRP)-mediated cross-linking. Surface plasmon resonance (SPR) measurements show that the AlgS-Ph hydrogel has an affinity for FGF-2 in accordance with its degree of sulfation. Conditions for the preparation of the AlgS-Ph hydrogel, including HRP and H_2_O_2_ concentrations, are optimized so that the hydrogel can be used as an injectable drug carrier. The hydrogel shows no cytotoxicity when using 10T1/2 cells as a model cell line. The angiogenesis assay shows that FGF-2 released from the AlgS-Ph hydrogel promotes the formation of blood vessels. These results indicate that the AlgS-Ph hydrogel is a suitable candidate for the FGF-2 carrier.

## 1. Introduction

The ultimate goal of tissue engineering is to regenerate damaged tissue and restore its biological function. In the regeneration of damaged tissues, numerous growth factors are known to promote cell proliferation, differentiation, and secretion of the extracellular matrix (ECM) [1,2,3]. One growth factor, fibroblast growth factor-2 (FGF-2), has mitogenic effects on fibroblasts, smooth muscle cells, and vascular endothelial cells and plays important roles in angiogenesis, tissue regeneration, and embryonic development [4,5]. Angiogenesis is especially critical for the successful regeneration of damaged tissue. Therefore, the effective use of FGF-2 for angiogenesis is an important issue. In the body, FGF-2 is stabilized by heparin and heparan sulfate, which are major components of the ECM, through intermolecular interaction. [4,6,7]. Owing to these components, endogenous FGF-2 is effectively delivered to receptors on the cell surface [8,9]; however, exogenously delivered FGF-2 is vulnerable to enzymatic degradation, thermal degradation, and diffusion, resulting in its inefficient delivery to damaged tissues [10,11]. Hence, methods for local and continuous FGF-2 delivery are required for its effective application to tissue regeneration.

Various methods have been considered for the localized and sustained delivery of FGF-2. For the localized and sustained delivery of FGF-2, hydrogel delivery systems have been studied [12,13]. Owing to advantages such as tunable mechanical properties and interaction with encapsulated FGF-2, hydrogels serve as a platform for the controlled release of FGF-2 [14,15,16,17]. Among various hydrogels, hydrogels consisting of alginate and its derivatives are frequently used materials. Alginate is a biocompatible polysaccharide composed of *β*-D-mannuronic acid and *α*-L-glucuronic acid [18]. It is well-known that an aqueous solution containing alginate forms a thermally stable hydrogel in the presence of divalent cations [15,18,19]. Among methods using hydrogels made from alginate derivatives, the loading of FGF-2 onto sulfated alginate or heparin-modified alginate hydrogels has attracted attention [20,21]. In this approach, the sulfation or heparin modification of alginate improves the effectiveness of FGF-2 delivery relative to unmodified alginate. However, several issues remain, including the low stability of sulfated alginate hydrogels in physiological conditions and the heterogeneity of heparin monosaccharide arrangements and sulfation patterns [22,23].

**Figure 1:**
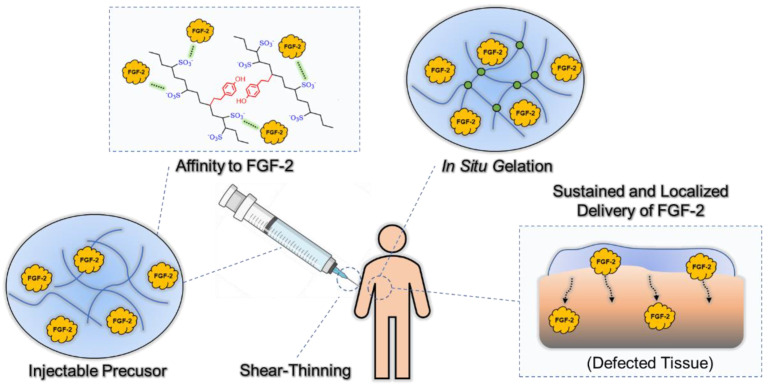
Concept of this study.

Among various hydrogels, injectable and in situ gellable hydrogels have also attracted attention [24,25]. The hydrogel precursor should be injectable solutions before administration, but the solutions should rapidly become hydrogel after injection into the body [26,27]. The most advantageous property of these material systems is their minimally invasive implant procedure. From these advantages, injectable hydrogel with an affinity for FGF-2 is a promising candidate for FGF-2 carrier. In this study, we prepared a hydrogel of phenol-grafted alginate sulfate (AlgS-Ph) for use as an injectable FGF-2 carrier (Figure 1). AlgS-Ph was expected to have FGF-2 affinity as a consequence of its sulfate groups. The hydrogel was prepared via a mild cross-linking reaction that used horseradish peroxidase (HRP) and H_2_O_2_ [28]. The gelation time was optimized by tuning the concentrations of HRP and H_2_O_2_. First, sulfate and phenolic hydroxyl groups were introduced into alginate, using chlorosulfonic acid and tyramine, respectively. The resulting AlgS-Ph hydrogels were characterized, and their affinity for FGF-2 was measured by using surface plasmon resonance (SPR). The mechanical properties of the hydrogels, including stiffness, swelling ratio, and gelation time, were measured. The cytocompatibility of the hydrogels was confirmed by culturing 10T1/2 cells. Finally, a chick chorioallantoic membrane (CAM) assay was performed to evaluate angiogenesis by FGF-2 released from the AlgS-Ph hydrogel.

## 2. Results and Discussion

### 2.1. Synthesis and Characterization of AlgS-Ph

#### 2.1.1. Synthesis of AlgS-Ph

**Figure 2:**
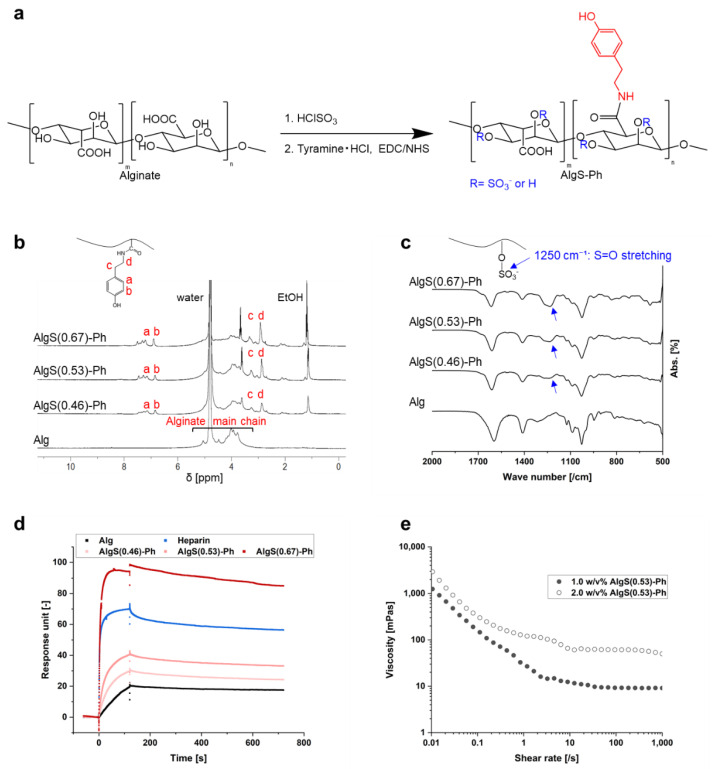
Characterization of AlgS-Ph. (**a**) Reaction scheme for preparing AlgS-Ph. (**b**) ¹H NMR spectra of AlgS-Ph (D₂O, 400 MHz). (**c**) FTIR spectra of AlgS-Ph. (**d**) SPR analysis of alginate (Alg), AlgS-Ph at various degrees of sulfation and heparin. FGF-2 is immobilized on the sensor surface. (**e**) Shear-rate–viscosity profiles of solutions containing different concentrations of AlgS(0.53)-Ph (1.0 *w*/*v*% and 2.0 *w*/*v*%) at 25 °C.

Sulfate and phenolic hydroxyl groups were introduced into alginate, using chlorosulfonic acid (HClSO_3_) and tyramine hydrochloride, respectively (Figure 2a). The synthesis of AlgS-Ph was confirmed by ^1^H NMR and FTIR spectroscopy. In the ^1^H NMR spectra, peaks attributable to phenol groups (6.7–7.2 ppm) were found only for AlgS-Ph (Figure 2b). The FTIR spectrum of AlgS-Ph showed absorbance peaks around 1250 cm^−1^, which are attributable to S=O stretching vibrations (Figure 2c). These observations are consistent with previous reports of tyramine- and sulfate-modified alginate [22,29,30].

AlgS-Ph with varying degrees of sulfation were prepared; using colloidal titration (Supplementary Materials Figure S1) [31], the degrees of sulfation were determined to be 0.46, 0.53, and 0.67 per monosaccharide unit and denoted as AlgS(0.46)-Ph, AlgS(0.53)-Ph, and AlgS(0.67)-Ph, respectively. The colloidal titration indicated that it is possible to adjust the number of sulfate groups by changing the concentration of HClSO_3_. The preparation of sulfated polysaccharides with a predetermined number of sulfate groups would be useful compared to heparan sulfate, in which the degree of sulfation often varies depending on origin and lots.

#### 2.1.2. Binding of FGF-2 to AlgS-Ph

The affinity of AlgS-Ph for FGF-2 was measured by SPR. As shown in Figure 2d, the FGF-2 affinity of AlgS-Ph is stronger than that of unmodified alginate. In addition, the affinity of AlgS-Ph for FGF-2 increased with increasing degree of sulfation; AlgS(0.67)-Ph had a stronger affinity than the heparin positive control. This result is consistent with previous studies, which show that the FGF-2 affinity is increased by the introduction of sulfate groups into alginate and hyaluronan [20]. This is due to electrostatic interactions between the sulfate groups and FGF-2 [32,33]. It is therefore assumed that the release profile of FGF-2 from the AlgS-Ph hydrogel can be controlled by adjusting the degree of sulfation. To avoid side effects when using AlgS-Ph with a too-high affinity in vivo, we used AlgS(0.53)-Ph, which has slightly weaker affinity to FGF-2 compared to heparin, in the following experiments.

#### 2.1.3. Viscoelasticity of AlgS-Ph Solutions

To investigate the injectability of the AlgS-Ph solution, shear-rate–viscosity profiles of aqueous solutions containing different concentrations of AlgS-Ph (1.0 *w*/*v*% and 2.0 *w*/*v*%) were measured by using a rheometer. As shown in Figure 2e, phosphate-buffered saline containing 2.0 *w*/*v*% AlgS(0.53)-Ph has a higher viscosity than a 1.0 *w*/*v*% solution. The viscosity decreased with increasing shear rate, indicating typical shear-thinning properties.

An injectable drug carrier requires a solution with a low enough viscosity to be extruded with a syringe, and the injected solution must remain at the target site until gelation. The shear-thinning properties of the AlgS-Ph solution are therefore suitable for an injectable drug carrier. The observed shear-thinning properties may be explained by the disentanglement and alignment of polymer chains [34].

### 2.2. Mechanical Properties of AlgS-Ph Hydrogel

#### 2.2.1. Gelation Time

**Figure 3:**
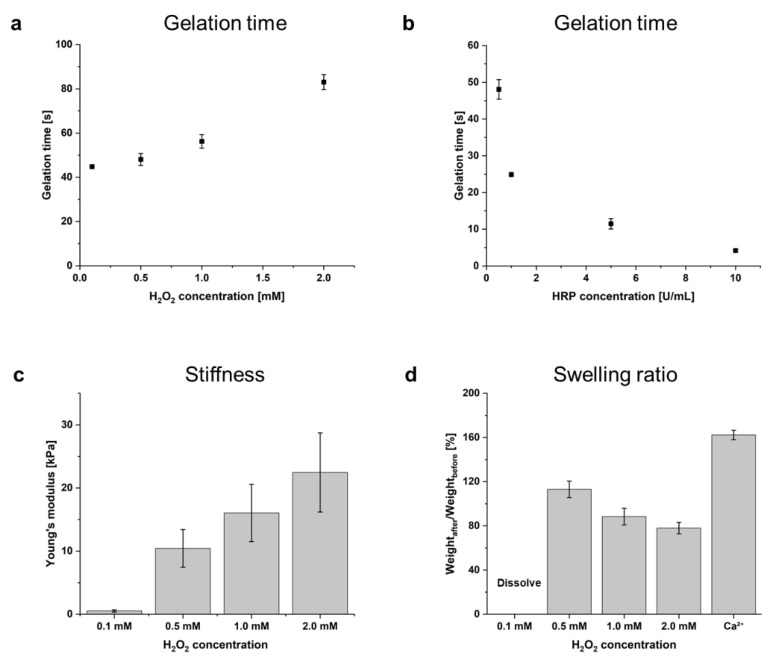
Dependence of gelation time on concentrations of (**a**) H_2_O_2_ at 2.0 *w*/*v*% AlgS(0.53)-Ph and 0.5 U/mL HRP, and (**b**) HRP at 2.0 *w*/*v*% AlgS(0.53)-Ph and 0.5 mM H_2_O_2_. Bars: mean ± SD (*n* = 4). (**c**) Dependence of Young’s modulus on concentrations of H_2_O_2_ (0.1, 0.5, 1.0, 2.0 mM) at 2.0 *w*/*v*% AlgS(0.53)-Ph and 0.5 U/mL HRP. (**d**) Swelling ratio of enzymatically cross-linked AlgS(0.53)-Ph hydrogels and ionically cross-linked AlgS-Ph hydrogel. Bars: mean ± SD (*n* = 5).

The effect of various HRP and H_2_O_2_ concentrations on the in situ gelation of AlgS-Ph was investigated. The gelation time of solutions of 2.0 *w*/*v*% AlgS(0.53)-Ph containing various concentrations of HRP (0.5, 1.0, 5.0, and 10 U/mL) and H_2_O_2_ (0.1, 0.5, 1.0, and 2.0 mM) was measured. As shown in Figure 3a,b, the gelation time was greater at lower H_2_O_2_ and higher HRP concentrations; a solution containing 2.0 *w*/*v*% AlgS(0.53)-Ph with 0.5 U/mL HRP and 0.5 mM H_2_O_2_ took approximately 48.1 ± 2.7 s to prepare a hydrogel.

For use as an injectable FGF-2 carrier, the gelation time of the hydrogel needs to be adjustable. Our observation that the gelation time can be controlled by changing the HRP and H_2_O_2_ concentrations is consistent with a previous report [28]. The decrease in gelation time with increased HRP concentrations (Figure 2b) can be rationalized by reaction stoichiometry. On the other hand, the increase in gelation time with increasing H_2_O_2_ concentrations (Figure 2a) is thought to be due to the inactivation of HRP by H_2_O_2_ oxidation [35]. Since high H_2_O_2_ concentrations may cause cytotoxicity [36], appropriate reaction conditions should be selected for each application. Photographs of injected hydrogel in PBS and cross-linked hydrogel are shown in Supplementary Materials Figure S2.

#### 2.2.2. Mechanical Properties of Hydrogel

To investigate the dependence of hydrogel stiffness on HRP and H_2_O_2_ concentrations, Young’s modulus of hydrogels prepared with different concentrations of H_2_O_2_ (0.1. 0.5, 1.0, and 2.0 mM) at 2.0 *w*/*v*% AlgS(0.53)-Ph and 0.5 U/mL HRP were estimated. Stress–strain curves were obtained from compression tests for the range of 1–10% strain (Figure 3c). The Young’s modulus of AlgS(0.53)-Ph increased from 0.5 ± 0.2 kPa at 0.1 mM H_2_O_2_ to 22.5 ± 6.3 kPa at 2.0 mM H_2_O_2_.

It is known that foreign-body reactions can occur after the implantation of biomaterials [37]. The severity of the foreign-body reaction is dependent on the difference in stiffness between the host tissue and the implanted material [37]. Because the stiffness of living tissues varies widely [38], the stiffness of AlgS-Ph hydrogels must be adjusted to each implant site. In the HRP-mediated cross-linking reaction, it is possible to control the cross-linking density, and therefore the hydrogel stiffness, by changing the H_2_O_2_ concentration [28]. As shown in Figure 3c, the stiffness of the hydrogel was adjusted by changing the H_2_O_2_ concentration, even in the case of AlgS-Ph. It is therefore expected that the stiffness of AlgS-Ph hydrogels can be tuned for each implant site.

#### 2.2.3. Swelling Ratio

The phenol, sulfate, and carboxylate groups of AlgS-Ph are potential cross-linking sites. To investigate the stability of AlgS-Ph hydrogels containing dual cross-linking sites, the swelling ratio of each hydrogel was investigated (Figure 3d). AlgS(0.53)-Ph hydrogels were prepared by dual (enzymatic and/or ionic) cross-linking, and the swelling ratios (*w*/*w*) were measured by soaking the hydrogels in simulated body fluid (SBF) for 3 days [39]. Ionically cross-linked AlgS(0.53)-Ph hydrogels swelled to 162.3 ± 4.3% of their initial volume. Enzymatically cross-linked AlgS-Ph hydrogels prepared with 0.1 mM H_2_O_2_ largely swelled and dissolved. However, in the case of AlgS-Ph hydrogels prepared with 0.5–2.0 mM H_2_O_2_, the gel samples maintained their shape, and their swelling ratio decreased from 113.0 ± 7.5% at 0.5 mM H_2_O_2_ to 78.0 ± 5.2% at 2.0 mM H_2_O_2_.

Because of their sulfate groups, ionically cross-linked sulfated alginate hydrogels are known to be more susceptible to swelling than unmodified alginate hydrogels [22]. To achieve drug release over a longer period of time (hours to days), the low stability of sulfated alginate hydrogels in vivo needs to be addressed. Enzymatically cross-linked AlgS-Ph hydrogels (using 0.5–2.0 mM H_2_O_2_) had lower swelling ratios than the ionically cross-linked hydrogels. With 0.5 mM and 1.0 mM H_2_O_2_, the resultant AlgS-Ph hydrogels showed little change from their initial volumes (113 ± 8% and 88 ± 8%, respectively). In the case of 1.0 mM H_2_O_2_, the shrinkage is thought to be caused by ionic interactions between the carboxylate groups of AlgS-Ph and divalent cations, such as Mg^2+^ and Ca^2+^ in SBF. Simultaneous swelling occurs due to the release of unreacted AlgS-Ph from the hydrogel. These adverse phenomena are thought to induce a smaller change in the volume of the hydrogel at high H_2_O_2_ concentrations. Regarding the degradation performance of AlgS-Ph hydrogels, AlgS-Ph hydrogel immersed in PBS containing 1 mg/mL alginate lyase was completely degraded within 12 h (Supplementary Materials Figure S3).

### 2.3. Cytocompatibility

**Figure 4:**
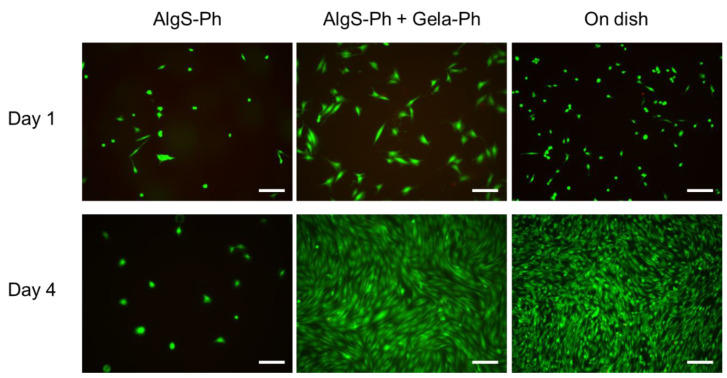
Merged micrographs of 10T1/2 cells cultured on hydrogels consisting of AlgS-Ph alone or AlgS-Ph and Gela-Ph together. Live and dead cells were stained with Calcein-AM/PI (green/red), respectively. Scale bars: 200 µm.

To evaluate the cytocompatibility of the AlgS-Ph hydrogel, 10T1/2 cells were cultured on hydrogels prepared from AlgS(0.53)-Ph alone or on AlgS(0.53)-Ph and phenol-grafted gelatin (Gela-Ph). Gela-Ph is known to have cell-adhesive properties [40]. As shown in Figure 4, the majority of 10T1/2 cells adhered to hydrogels containing both AlgS(0.53)-Ph and Gela-Ph, as well as to the dish on the day after seeding. However, fewer 10T1/2 cells adhered to the hydrogels containing AlgS(0.53)-Ph alone. The cells adhering to AlgS(0.53)-Ph hydrogel had spherical morphologies and did not proliferate, whereas those adhering to AlgS(0.53)-Ph and Gela-Ph hydrogel elongated and proliferated. The cells on the AlgS(0.53)-Ph and Gela-Ph hydrogel had larger widths than those on the dish.

Because the cells elongated and proliferated on the mixed hydrogel, the lack of elongation and proliferation on the AlgS(0.53)-Ph hydrogel is not due to its cytotoxicity but to its poor cellular adhesiveness. Moreover, the results indicate that AlgS-Ph has good cytocompatibility and is applicable as an FGF-2 carrier. The low cellular adhesiveness of AlgS-Ph is due to its hydrophilicity and anionic nature, which both prevent the adsorption of proteins necessary for cell adhesion [41]. The wider shape of cells on the hydrogel is due to the lower stiffness of the hydrogel compared to that of the cell culture dish. These properties are consistent with those reported previously [42,43].

### 2.4. CAM Assay

**Figure 5:**
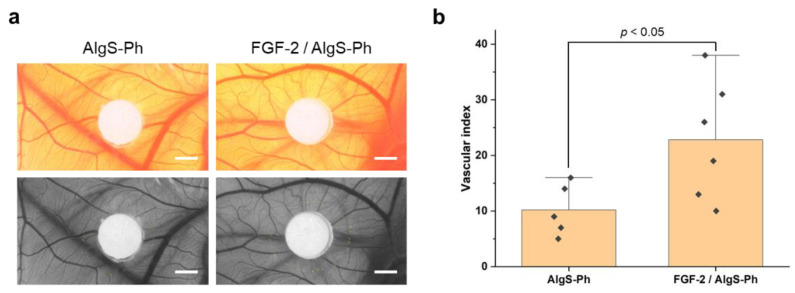
Evaluation of angiogenesis using a chick embryo model. (**a**) Original and 16-bit micrographs show the blood vessels near each filter paper; put on the chick embryo treated with AlgS-Ph hydrogel alone or AlgS-Ph hydrogel containing 50 ng of FGF-2 on two days after implantation. Scale bars: 2 mm. (**b**) Vascular index of each sample. Bars: mean ± SD (*n* = 5 or 6).

To evaluate the performance of AlgS-Ph hydrogel as an FGF-2 carrier, an angiogenesis assay was performed by using CAM. Filter papers with hydrogels containing 2.0 *w*/*v*% AlgS(0.53)-Ph alone and 2.0 *w*/*v*% AlgS(0.53)-Ph + 5 ng/µL FGF-2 were put on CAM. The vascular index was calculated from the micrographs of blood vessels on CAM by the method previously reported [44]. Figure 5a shows the newly formed blood vessels around each filter paper two days after the implantation. Around the filter paper with AlgS(0.53)-Ph hydrogel containing FGF-2, radially extended blood vessels were observed. Figure 5b indicates the vascular index for each sample. Compared with the AlgS(0.53)-Ph hydrogel alone, the AlgS-Ph hydrogel containing FGF-2 showed a higher number of radially extended blood vessels (*p* < 0.05, *t*-test).

A CAM assay was performed to confirm whether the FGF-2 released from hydrogel retains its biological activity or not [45,46]. The larger number of blood vessels around the AlgS-Ph hydrogel containing FGF-2 than that around the AlgS-Ph hydrogel alone is because of the effect of the released FGF-2 [47,48]. This result suggests that HRP-mediated cross-linking reaction is mild enough to retain the biological activities of FGF-2 encapsulated in the AlgS-Ph hydrogel, and the FGF-2 released from hydrogel promotes angiogenesis. It is therefore concluded that the AlgS-Ph hydrogel can be used as an FGF-2 carrier.

## 3. Conclusions

Injectable and in situ gellable AlgS-Ph hydrogels were fabricated by tuning the concentrations of HRP and H_2_O_2_. The affinity of the hydrogels for FGF-2 was modulated by changing the degree of sulfation. The AlgS-Ph hydrogel displayed no obvious cytotoxicity, and 10T1/2 cells adhered to a mixed hydrogel consisting of AlgS-Ph and Gela-Ph. The CAM assay showed that FGF-2 released from AlgS-Ph hydrogels promotes angiogenesis. These results demonstrate the potential application of the AlgS-Ph hydrogel as an FGF-2 carrier.

## 4. Materials and Methods

### 4.1. Materials

Sodium alginate (100–200 mPa·s (1%)) was purchased from Kimica Co. (Tokyo, Japan). Tyramine hydrochloride was purchased from Combi-Blocks (San Diego, CA, USA). Water-soluble carbodiimide (WSCD) was purchased from Peptide Institute (Osaka, Japan). *N*-hydroxysuccinimide (NHS), aqueous hydrogen peroxide (H_2_O_2_, 31 *w*/*w*%), 2- (*N*-morpholino)ethanesulfonic acid (MES), heparin sodium, formamide (98.5%), N/400 potassium polyvinyl sulfate solution (PVSK) solution, N/200 glycol chitosan (Gch) solution, toluidine blue indicator solution, 2-[4-(2-hydroxyethyl)-1-piperazinyl] ethanesulfonic acid (HEPES), and catalase from bovine liver were purchased from Fujifilm Wako Pure Chemical Industries (Osaka, Japan). HRP (68 U/mg) was purchased from Toyobo Co. (Osaka, Japan). Chlorosulfonic acid (HClSO_3_, >97%) was purchased from Tokyo Chemical Industry (Tokyo, Japan). Mouse fibroblast 10T1/2 cells were obtained from Riken Cell Bank (Ibaraki, Japan) and were grown in Dulbecco’s Modified Eagles Medium (DMEM, Nissui, Tokyo, Japan) supplemented with 10 *v*/*v*% fetal bovine serum in a 5% CO_2_ incubator. Human recombinant FGF-2 was purchased from DS Pharma Biomedical (Osaka, Japan).

### 4.2. Synthesis of AlgS-Ph

The sulfation of alginate was performed as previously reported [49]. The reaction was tuned by varying the concentration of HClSO₃; three degrees of sulfation were afforded (Table 1). Under a nitrogen atmosphere at 60 °C, three concentrations (1.75 *v*/*v*%, 2.25 *v*/*v*%, and 2.75 *v*/*v*%) of HClSO_3_ were added to formamide in a total reaction volume of 40 mL. Next, 1 g of alginate powder was added, and the reaction mixture was stirred constantly for 2.5 h. The product was precipitated with a 4-fold (*v*/*v*) volume of cold acetone and isolated by centrifugation at 3500 rpm for 1 min. The precipitate was dissolved in deionized water, and the pH was adjusted to 7.0, using a 5 M NaOH aqueous solution. The solution was dialyzed against aqueous 100 mM NaCl solution overnight and then dialyzed against deionized water for 6 h and freeze-dried. The incorporation of tyramine into the sulfated alginate was carried out as previously reported [39]. AlgS (1 g) was dissolved in 71 mL MES-buffered solution (pH 6.0). Tyramine hydrochloride (0.848 g), NHS (0.123 g), and WSCD (0.326 g) were added sequentially, and the reaction mixture was stirred for 20 h at room temperature. Then the pH was adjusted to 8.6 with 1 M NaOH solution. The resulting polymer was precipitated with excess acetone and washed three times with 80 *v*/*v*% ethanol and once with 100 *v*/*v*% ethanol.

### 4.3. Characterization

^1^H NMR spectra were measured by using a JNM-ECS400 instrument (JEOL, Tokyo, Japan), and FTIR spectra were measured by using an FT/IR-4100 instrument (JASCO, Tokyo, Japan). The degree of sulfation per monosaccharide was measured by colloidal titration as previously reported [31], and details are shown in Supplementary Materials Figure S1. AlgS-Ph (5 mg) was dissolved in 5 mL of pure water, and the pH of the solution was adjusted to 1.0. Then 5 mL of Gch solution and three drops of toluidine blue were added sequentially. N/400 PVSK solution was added dropwise until the color of the solution changed from blue to purple.

### 4.4. Affinity to FGF-2

To evaluate the interaction between FGF-2 and AlgS-Ph, an SPR analysis was performed by using a Biacore^TM^ T200 instrument (GE Healthcare, Uppsala, Sweden). Measurements were performed at 25 °C, using HBS-EP buffer (10 mM HEPES, 150 mM NaCl, 3 mM EDTA, 0.005 *v*/*v*% SP-20, pH 7.4) as a running buffer and sodium acetate solution (pH 4.0) as a regenerative buffer, respectively. Immobilization of FGF-2 on the surface of the CM5 sensor chip (Cytiva, Tokyo, Japan) was performed by amide coupling, using NHS and WSCD. During the measurement, HBS-EP buffer with 10 µg/mL of alginate, AlgS(0.46)-Ph, AlgS(0.53)-Ph, AlgS(0.67)-Ph, and heparin were injected into the channel (association time, 2 min; dissociation time, 10 min).

### 4.5. Shear-Rate–Viscosity Measurement

Shear-rate–viscosity profiles of AlgS(0.53)-Ph solutions of different concentrations (1.0 *w*/*v*%, 2.0 *w*/*v*%) were measured with a rheometer (HAAKE MARS III, Thermo Fisher Scientific, Waltham, MA, USA). The instrument was equipped with a parallel plate of a 20 mm radius. Measurements were performed with a 0.5 mm gap at 20 °C.

### 4.6. Gelation-Time Measurement

Gelation times of AlgS(0.53)-Ph solutions containing different concentrations of H_2_O_2_ (0.1, 0.5, 1.0, and 2.0 mM) and HRP (0.5, 1.0, 5.0, and 10 U/mL) were determined. An AlgS(0.53)-Ph solution (200 µL) and an HRP solution (50 µL) were added to each well of a 48-well plate. Subsequently, 50 µL of H_2_O_2_ solution was added to each well with constant stirring. The gelation time was defined as the time from addition of H_2_O_2_ solution to the formation of a hydrogel.

### 4.7. Stiffness Measurement

The stiffness of AlgS(0.53)-Ph hydrogels was evaluated by measuring the repulsion forces toward compression, using a tabletop materials tester (EZ-test, Shimadzu, Kyoto, Japan). AlgS(0.53)-Ph (3.0 *w*/*v*%), HRP (3.0 U/mL), and H_2_O_2_ (12, 6, 3, 0.5 mM) solutions were cooled to 4 °C. AlgS(0.53)-Ph solution (800 µL) and HRP solution (200 µL) were mixed in 1.5 mL Eppendorf tubes. Subsequently, 200 µL of H_2_O_2_ solution was added, and then the solutions were mixed with a vortex mixer for 3 s. The resulting cylindrical hydrogels were pulled out of the tubes and cut at 3 mm intervals to obtain hydrogel discs. The obtained discs were used to measure the repulsion forces (5 mm diameter) toward compression (6 mm/min). Young’s modulus was estimated from the slope of stress–strain curves in the range of 1–10% strain.

### 4.8. Swelling-Ratio Measurement

The swelling ratio of AlgS(0.53)-Ph hydrogels prepared by different cross-linking methods was evaluated by measuring the ratio of mass change in SBF. Enzymatically cross-linked hydrogels were obtained by using the same method as described in Section 4.7. To obtain ionically cross-linked hydrogels, 5 mL of AlgS(0.53)-Ph solution (2.0 *w*/*v*%) was added to dialysis membrane tubes (MWCO: 12–15 kDa) and hung in 40 mL of a solution containing 50 mM of calcium chloride for 30 min. After preparation, the hydrogel was cut into the same shape as the enzymatically cross-linked hydrogel discs. The mass of the obtained hydrogel discs was measured, and the discs were soaked in SBF for 3 days and measured again. The swelling ratio was calculated as weight_after_/weight_before_ × 100 (%).

### 4.9. Cytocompatibility

The cytocompatibility of AlgS(0.53)-Ph was evaluated by using AlgS(0.53)-Ph hydrogels and 10T1/2 cells. Solutions containing 2.0 *w*/*v*% AlgS(0.53)-Ph alone or both 2.0 *w*/*v*% AlgS(0.53)-Ph and 1.0 *w*/*v*% Gelatin-Ph, and 5 U/mL HRP were poured into a 6-well plate at 1 mL/well. Next, the plate was put into a plastic container, and air containing H_2_O_2_ (obtained by bubbling air through 0.5 M H_2_O_2_ solution) was allowed to flow into the plastic container for 30 min. The resulting hydrogel sheets were soaked in a 2.0 mL solution of 2000 U/mL of catalase for 20 min. Subsequently, 10T1/2 cells were suspended in DMEM and added to each well at 2.0 × 10^4^ cells/well. On days 1 and 4, live and dead cells were stained with Calcein-AM and PI, respectively, before observing with a fluorescence microscopy.

### 4.10. CAM Assay

To evaluate the performance of the AlgS(0.53)-Ph hydrogel as an FGF-2 carrier, an angiogenesis assay was performed by using CAM. The CAM assay was carried out as previously reported [50]. First, 10 µL of a solution containing 2.0 *w*/*v*% AlgS(0.53)-Ph + 0.5 U/mL HRP + 5 ng/µL FGF-2 or 2.0 *w*/*v*% AlgS(0.53)-Ph + 0.5 U/mL HRP was poured on the filter paper with a 4 mm diameter. Subsequently, solution-soaked filter papers were put in the plastic container and exposed to the air containing H₂O₂ that was obtained by bubbling air in 0.5 M H₂O₂ solution for 1 min. Prepared filter papers coated with hydrogels were put on the CAM on embryonic development day 8. CAM was incubated in the incubator (37 °C, 80%RH). The blood vessels around the filter paper were observed again on embryonic development day 10. To quantitatively evaluate angiogenesis, the vascular index was measured by the method previously reported [44].

## Figures and Tables

**Table 1:** Concentrations of alginate and HClSO_3_ and reaction time for preparing of AlgS-Ph.

Name	Alginate (*w*/*v*%)	HClSO_3_ (*v*/*v*%)	Reaction Time (h)
AlgS(0.46)-Ph	2.5	1.75	2.5
AlgS(0.53)-Ph	2.5	2.25	2.5
AlgS(0.67)-Ph	2.5	2.75	2.5

## Data Availability

All data generated or analyzed during this study are included in this published article and its supplementary files.

## Supplementary Materials

The following are available online at https://www.mdpi.com/article/10.3390/gels8120818/s1, Figure S1: Colloidal titration. (a) The reaction to form polyion complex between polycation (glycol chitosan (Gch)) and polyanion (potassium polyvinyl sulfate (PVSK)). (b) schematic diagram of a titration; Figure S2: (a) AlgS(0.53)-Ph hydrogel dyed with green-color dye injected from 27G needle into PBS. (b) Cross-linked hydrogel; Figure S3: Degradation test of AlgS(0.53)-Ph hydrogel dyed with pink-color dye in PBS with or without 1 mg/mL alginate lyase. (Scale bar: 2 cm).
