# Supplementary material for: Phenol-Grafted Alginate Sulfate Hydrogel as an Injectable FGF-2 Carrier
Source: Gels. 2022 Dec 12;8(12):818. doi: 10.3390/gels8120818 (PMC9778324; doi:10.3390/gels8120818)
Supplement: Supplementary file 1 [file gels-08-00818-s001.zip › gels-2057175-supplementary.pdf]

gels

Article

Phenol-Grafted Alginate Sulfate Hydrogel as an Injectable FGF-2 Carrier

Ryota Goto [1], Masaki Nakahata [2,*] and Shinji Sakai [1,*]

[1] Department of Materials Engineering Science, Graduate School of Engineering Science, Osaka University, Toyonaka 560-8531, Japan
[2] Department of Macromolecular Science, Graduate School of Science, Osaka University, Toyonaka 560-0043, Japan
* Correspondence: nakahata@chem.sci.osaka-u.ac.jp (M.N.); sakai.shinji.es@osaka-u.ac.jp (S.S.)

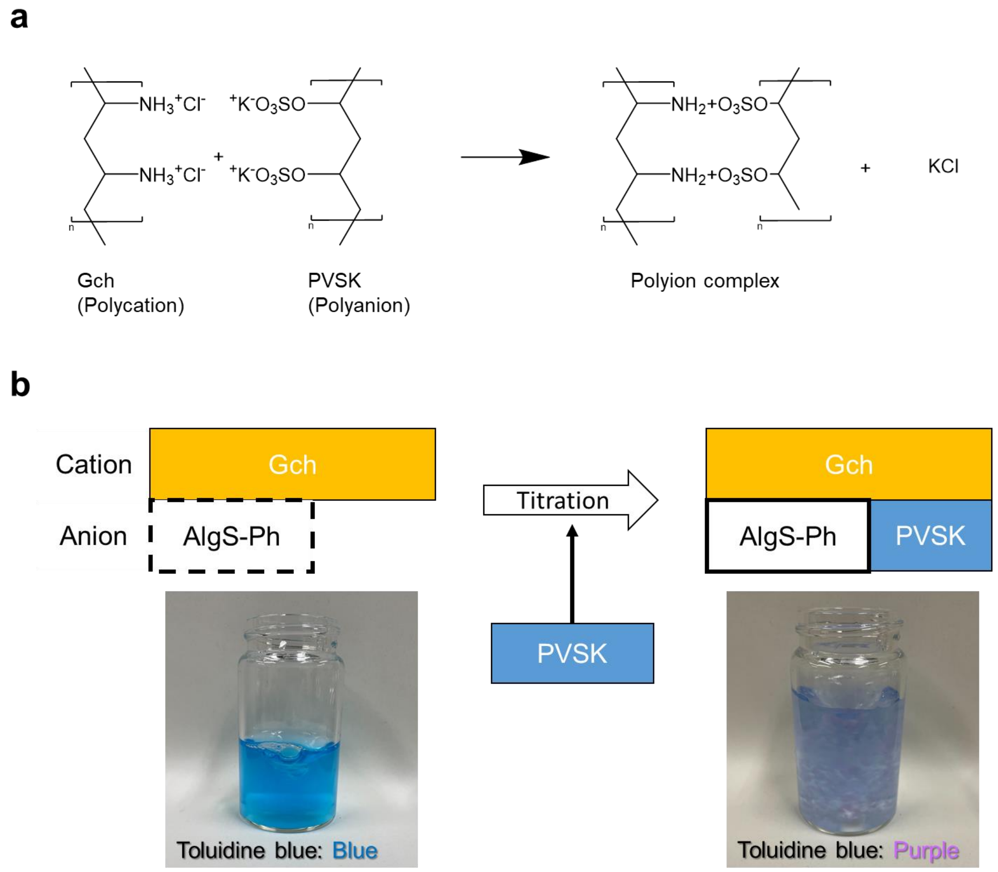

Figure S1. Colloidal titration. (a) The reaction to form polyion complex between polycation (glycol chitosan (Gch)) and polyanion (potassium polyvinyl sulfate (PVSK)). (b) schematic diagram of a titration.

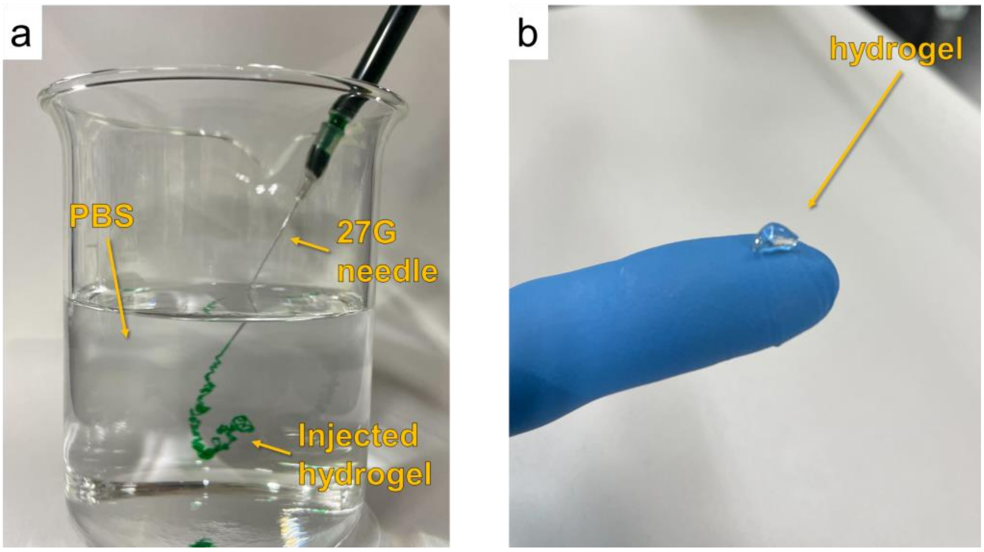

Figure S2. (a) AlgS(0.53)-Ph hydrogel dyed with green-color dye injected from 27G needle into PBS. (b) Cross-linked hydrogel

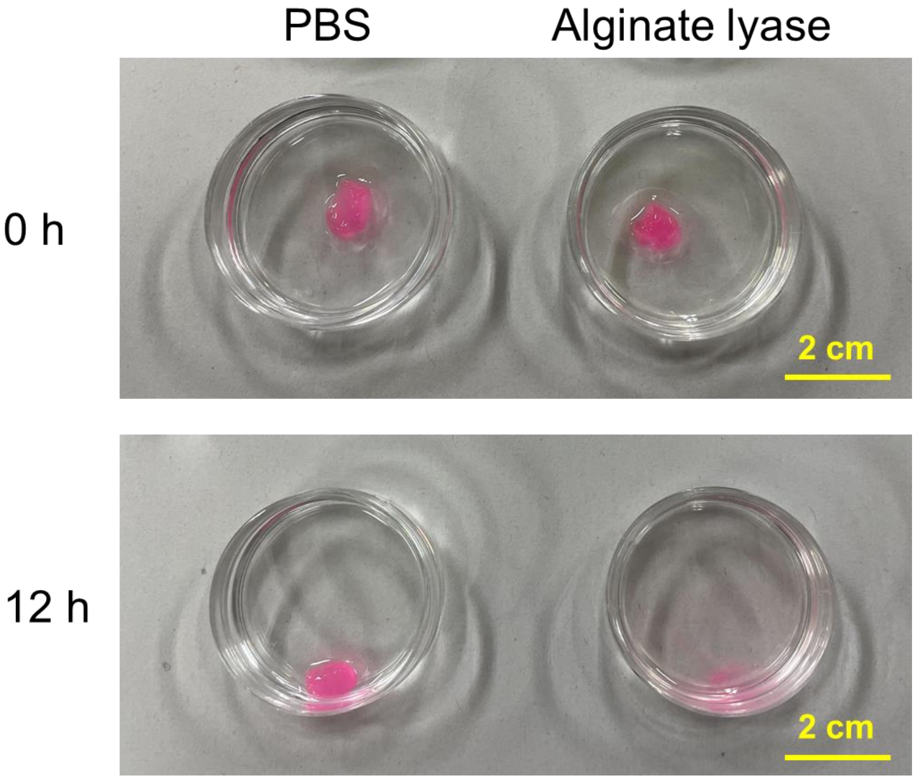

Figure S3. Degradation test of AlgS(0.53)-Ph hydrogel dyed with pink-color dye in PBS with or without 1 mg/mL alginate lyase. (Scale bar: 2 cm)
